# Calpain inhibition ameliorates scald burn-induced acute lung injury in rats

**DOI:** 10.1186/s41038-018-0130-3

**Published:** 2018-10-08

**Authors:** Peng-Ran Du, Hong-Ting Lu, Xi-Xiang Lin, Li-Feng Wang, Yan-Xia Wang, Xiao-Ming Gu, Xiao-Zhi Bai, Ke Tao, Jing-Jun Zhou

**Affiliations:** 1 Department of Physiology and Pathophysiology, Fourth Military Medical University, No. 169 Changle West Road, Xi’an, 710032 China; 2 Department of Biochemistry, Fourth Military Medical University, Xi’an, Shaanxi China; 3 Department of Pathology, Fourth Military Medical University, Xi’an, Shaanxi China; 4 Department of Burns and Cutaneous Surgery, Xijing Hospital, Fourth Military Medical University, Xi’an, Shaanxi China

**Keywords:** Acute lung injury, Burn, Calpain, Inflammation, Membrane skeleton proteins, Scald

## Abstract

**Background:**

The molecular pattern of severe burn-induced acute lung injury, characterized by cell structure damage and leukocyte infiltration, remains unknown. This study aimed to determine whether calpain, a protease involved in both processes, mediates severe burn-induced acute lung injury.

**Methods:**

Rats received full-thickness scald burns covering 30% of the total body surface area, followed by instant fluid resuscitation. MDL28170 (Tocris Bioscience), an inhibitor of calpain, was given intravenously 1 h before or after the scald burn. The histological score, wet/dry weight ratio, and caspase-3 activity were examined to evaluate the degree of lung damage. Calpain activity and its source were detected by an assay kit and immunofluorescence staining. The proteolysis of membrane skeleton proteins α-fodrin and ankyrin-B, which are substrates of calpain, was measured by Western blot.

**Results:**

Time-course studies showed that tissue damage reached a peak between 1 and 6 h post-scald burn and gradually diminished at 24 h. More importantly, calpain activity reached peak levels at 1 h and was maintained until 24 h, paralleled by lung damage to some extent. Western blot showed that the levels of the proteolyzed forms of α-fodrin and ankyrin-B correlated well with the degree of damage. MDL28170 at a dose of 3 mg/kg b. w. given 1 h before burn injury not only antagonized the increase in calpain activity but also ameliorated scald burn-induced lung injury, including the degradation of α-fodrin and ankyrin-B. Immunofluorescence images revealed calpain 1 and CD45 double-positive cells in the lung tissue of rats exposed to scald burn injury, suggesting that leukocytes were a dominant source of calpain. Furthermore, this change was blocked by MDL28170. Finally, MDL28170 given at 1 h post-scald burn injury significantly ameliorated the wet/dry weight ratio compared with burn injury alone.

**Conclusions:**

Calpain, a product of infiltrating leukocytes, is a mediator of scald burn-induced acute lung injury that involves enhancement of inflammation and proteolysis of membrane skeleton proteins. Its late effects warrant further study.

## Background

Acute lung injury is a serious complication of severe burns in patients with extensive deep burns and a burn area greater than 30% of the total body surface area [1]. Acute lung injury occurs in the first 24 to 48 h post-scald burn. The injury manifests as a compromised alveoli-capillary barrier, edema formation, leukocyte infiltration, and oxidative stress [2, 3]. The vicious pathophysiological alterations cause an increase in airway resistance and abnormality of gas exchange [4]. Some cases are hard to prevent with instant fluid resuscitation; such patients develop acute respiratory distress syndrome and burn shock, increasing the mortality risk [5]. To date, interventions to treat acute lung injury remain limited; this fact necessitates continued basic scientific research in this field to clarify the underlying mechanisms.

Calpains are Ca^2+^-dependent, nonlysosomal cysteine proteases. Currently, 15 genes have been described in the human genome, and calpain 1 is a well-studied and ubiquitously expressed isoform [6]. Under physiological conditions, controlled activation of calpain 1 results in the hydrolysis of target proteins, thereby initiating various biological events, including angiogenesis, cell adhesion, and memory formation [7, 8]. By contrast, hyperactivation of calpain 1 causes cell damage in pathological circumstances [9, 10]. The most common targets of attack are membrane skeleton proteins, especially α-fodrin and ankyrin-B [6, 9]. Both proteins are indispensable for maintaining cell shape and function [11–13]. Deficiency of these proteins is associated with defects in cell proliferation and adhesion [14], and has been implicated in human diseases, including arrhythmias and metabolic syndrome [15, 16]. Calpain 1 is also critical for the inflammatory response. It cleaves the inhibitor IκBα, thus activating inflammation transcription factor NF-κB. It promotes inflammatory cell infiltration and proinflammatory cytokine processing [17, 18]. Previous studies have demonstrated that cell structure damage and leukocyte infiltration are distinguishing features in scald burn-induced acute lung injury [2, 3, 19]. This prompted us to hypothesize that calpain mediates scald burn-induced acute lung injury.

Time course studies were performed to evaluate the severity of lung injuries and calpain activity after scald burn injury. The fragments levels of membrane skeleton proteins a-fodrin and ankyrin-B, which are substrates of calpain, were also examined. Then, we used MDL28170 (Tocris Bioscience) to block calpain activity in intact rats and determined its effects on acute lung injury. Concomitantly, we examined calpain 1 in the infiltrating leukocytes. Moreover, to evaluate its clinical relevance, we determined the effects of MDL28170 when given 1 h post-scald burn. Our data provided evidence that calpain, a product of infiltrating leukocytes, is a mediator of scald burn-induced acute lung injury. Its destructive actions involve enhancement of inflammation and proteolysis of membrane skeleton proteins.

## Methods

### Animal experimental protocol

Male Sprague-Dawley rats weighing 200 to 250 g provided by the Laboratory Animal Center of the Fourth Military Medical University were included in this study. The study was performed in accordance with the guidelines for the Care and Use of Laboratory Animals issued by Institutional Animal Care and Use Committee (IACUC) of the Fourth Military Medical University. This study was approved by Institutional Animal Care and Use Committee (IACUC) of Fourth Military Medical University (No. 20170304).

The severe scald burn injury model was prepared based on previous studies [20]. Briefly, the rats were anesthetized via i.p. injection of 40 mg/kg pentobarbital (Tocris Bioscience). After the dorsal and lateral back hair was shaved, the rats were placed in a wooden mold with an 8 × 5 cm opening corresponding to 30% of the total body surface area. The exposed area was then immersed in 92 °C water for 18 s to induce full-thickness skin damage. All the animals were quickly dried and then resuscitated with Ringer’s lactate solution (50 ml/kg b. w. i.p.). Subsequently, the rats received analgesia with buprenorphine (Tocris Bioscience) at a dose of 0.05 mg/kg b. w. (i.p.) every 8 h post-scald burn.

Time course studies were performed to evaluate calpain activity and the severity of lung injuries. The rats were sacrificed at 0.5, 1, 6, 12, and 24 h post-scald burn, with six animals for each time point respectively. For the sham group, six rats underwent identical procedures but were immersed in room temperature water. And then, the effects of the calpain inhibitor MDL28170 were evaluated. The rats were divided into the sham group, the sham with MDL28170 group, the burn injury group, and the burn injury with MDL28170 group, with six animals for each group. For the last group, MDL28170 at a dosage of 1 or 3 mg/kg b. w. was given intravenously through the tail vein 1 h before the scald burn. The drug was dissolved in dimethyl sulfoxide to a concentration of 50 mg/ml and brought up to a volume of 1 ml with saline. For the burn injury group, the same amount of dimethyl sulfoxide as that in the drug treatment group (15 μl diluted to 1 ml with saline) was injected. The treatment for the sham group and the following procedures for all the groups were the same as those for the first experiments. Examinations at 1 h post-scald burn were performed to compare the differences among groups. Moreover, to evaluate the effects of MDL28170 when given 1 h post-scald burn, the animals were randomly allocated to the burn injury group and the burn injury with MDL28170 group. Each group had six animals, and identical procedures were implemented for scald burn injury and drug delivery. The samples were examined at 6 h post-scald burn.

### Histological examination for lung injury and skin damage

Lung and dorsal skin tissues were fixed in 10% formalin, and paraffin-embedded sections were stained with hematoxylin and eosin. A pathologist who was unaware of the group assignment examined the sections and all the samples were examined by the same pathologist to ensure consistent judgment. To measure the degree of lung injuries, ten fields for two slides from each rat were analyzed under a × 200 field of microscope. A scoring system of 0–4 was used for each section based on the degree of septal thickening, congestion, hemorrhage, edema, and leukocyte infiltration. The scoring criteria were as follows: 0, normal appearance; 1, light; 2, moderate; 3, strong; and 4, intense and widespread abnormality, as described previously [4]. The mean value of each rat was used for statistical analysis.

### Measurement of lung wet/dry weight ratio

Left lung tissues were weighed to obtain the wet weight at the end of experiments. Then, the tissues were dried to a constant weight in an oven at 70 °C for 48 h. The wet/dry weight ratio was then calculated to quantify the magnitude of pulmonary edema.

### Calpain and caspase-3 activity assay

Calpain activity was detected with a kit using Suc-Leu-Leu-Val-Tyr-7-amino-4-methylcoumarin (AMC) as a substrate (Calbiochem), as described previously [9]. AMC was measured at excitation and emission wavelengths of 380 nm and 460 nm, respectively. Calpain activity was quantified by the amount of free AMC per minute per microgram of protein. The activity of caspase-3 was measured with Biovision’s kit using *N*-Acetyl-Asp-Glu-Val-Asp-*p*-nitroanilide as a substrate. Free *p*-nitroanilide was quantified using a microtiter plate reader at 405 nm, and the data were normalized to the sham group, assigned a value of 1 [9].

### Western blot

Total proteins were extracted with lysis buffer containing Tris-HCl (pH = 7.3) 50 mM, NaCl 150 mM, EDTA 5 mM, dithiothreitol 1 mM, 1% Triton X-100, 1% protease inhibitor cocktail, and 1% phosphatase inhibitor cocktail. The samples were centrifuged at 12000× rpm at 4 °C for 30 min. The supernatants were subjected to gel electrophoresis and were transferred onto polyvinylidene fluoride membranes. The membranes were incubated with primary antibodies against α-fodrin at 1:1000 (Enzo Life Sciences) and ankyrin-B at 1:200 (Santa Cruz) at 4 °C overnight, followed by horseradish peroxidase-conjugated secondary antibody for 2 h at room temperature. Finally, the membranes were detected by exposing to chemiluminescent horseradish peroxidase substrate and imaged with ChemiDOC XRS (Bio-Rad, Hercules, CA, USA). Blots were probed simultaneously for β-actin as a control for equal protein loading. Relative densitometry was analyzed using Image Lab (Bio-Rad Laboratories, Hearts, UK) [9].

### Double immunofluorescence histology

Paraffin-embedded tissue sections were stained with rabbit anti-calpain 1 at 1:100 (Cell Signaling Technology) and mouse anti-CD45 at 1:100 (Abcam) at 4 °C overnight. Then, the sections were incubated with tetramethylrhodamine-conjugated anti-rabbit and Alexa Fluor® plus 488-conjugated anti-mouse secondary antibodies (Molecular Probes), followed by nuclei counterstaining with 4′,6-diamidino-2-phenylindole. Finally, the sections were examined using a laser-scanning confocal microscope equipped with the FV10-ASW system (Olympus FV1000) [9]. Five fields for each slide from each rat are examined under a × 600 field of microscope, and the number of positive cells was counted. The mean value per field of each rat was used for statistical analysis.

### Statistical analysis

All values were expressed as mean ± standard error of mean (SEM). Statistical comparisons among several groups were carried out using one-way analysis of variance followed by Tukey’s test. Student’s *t* tests were performed to compare the differences between two groups. Data were analyzed with GraphPad Prism (GraphPad software Inc., La Jolla, CA, USA). A two-tailed *p* < 0.05 was accepted as statistically significant.

## Results

### Scald burn caused acute lung injury and an increase in calpain activity

**Fig. 1:**
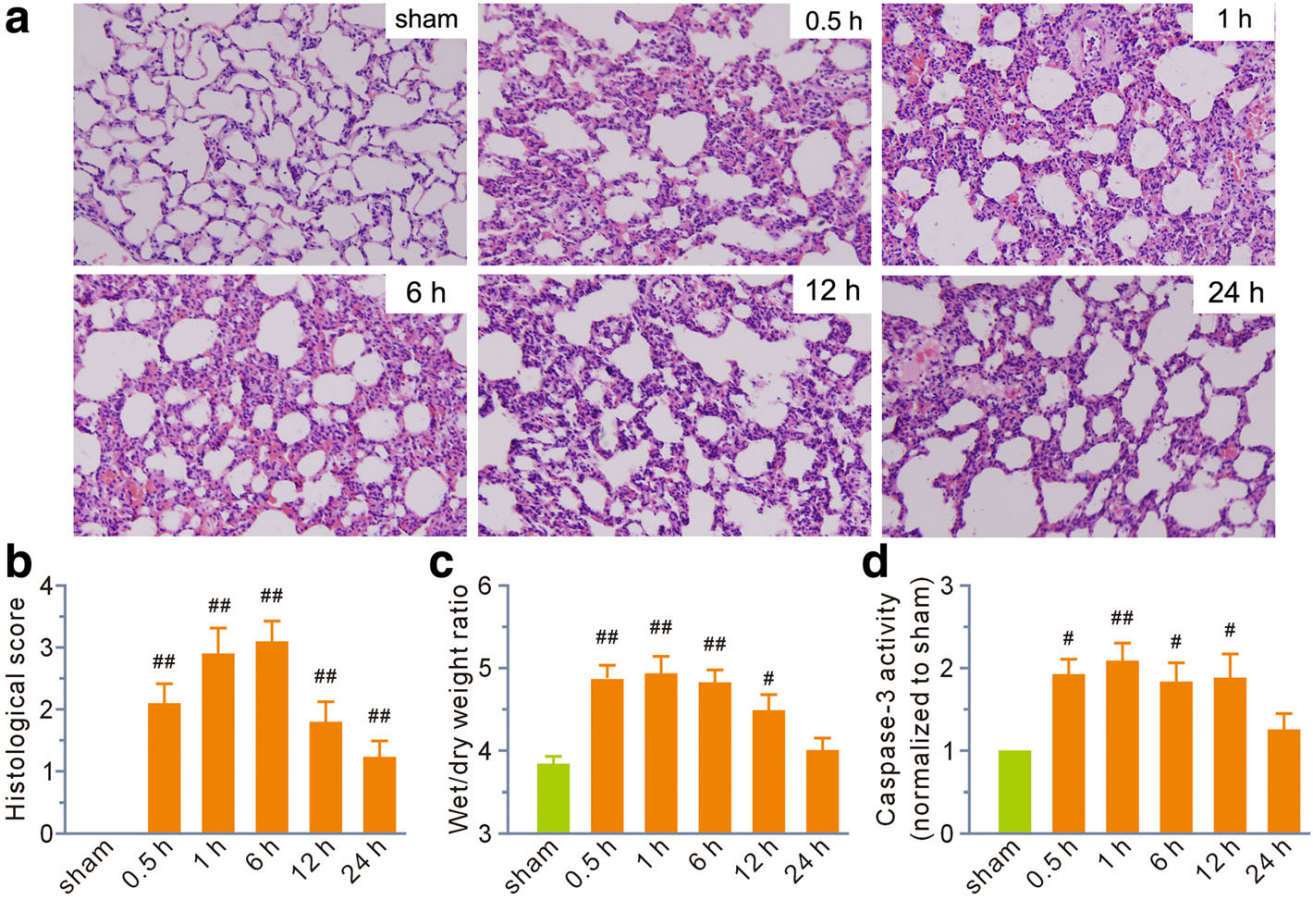
Scald burn caused extensive damage to the lung during the first 24 h following injury. **a** Representative hematoxylin and eosin staining showing morphological changes in lung tissue (magnification, × 200). 0.5 h, 1 h, 6 h, 12 h, and 24 h represented the time points for sampling after burn injury. **b**, **c** Grouped results of histology scores and wet to dry weight ratios, respectively. **d** Grouped results of caspase-3 activity. The values were normalized to the sham group, which was assigned a value of 1. Each bar represents the mean ± SEM; *n* = 6 rats. ^#^*P* < 0.05, ^##^*P* < 0.01 vs sham

All animals survived the 24h experimental period. Histological examination revealed that tissue damage appeared at 0.5 h post-scald burn and reached a peak between 1 and 6 h. Lung tissue showed a trend toward a return to normal at 24 h, although it remained damaged (Fig. 1a). Typical histopathological changes in lung tissue included prominent septal thickening, fusion of alveolar septa, congestion, hemorrhage, interstitial edema, enhanced migration of circulating leukocytes into the interstitial spaces, and cellular debris in the alveoli. Histological scoring was calculated as an index to assess overall tissue injury. The data showed that scald burn increased the value markedly at all the indicated time points (Fig. 1b).

The lung wet/dry weight ratios at 0.5, 1, 6, and 12 h post-scald burn were significantly higher than those in the sham group, and the values recovered to normal at 24 h (Fig. 1c). Similarly, the activity of caspase-3, a pro-apoptotic protein, rapidly increased at 0.5 h and reached a peak at 1 h, gradually returning to normal at 24 h (Fig. 1d).

**Fig. 2:**
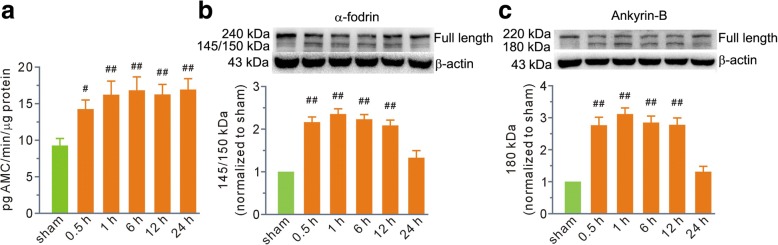
Scald burn increased calpain activity and degradation of its substrates α-fodrin and ankyrin-B in lung tissue. **a** Grouped results of calpain activity. **b**, **c** Top, representative blots for full-length α-fodrin and ankyrin-B, as well as their 145/150- and 180-kDa fragments. Bottom, grouped results of the densitometric analysis. β-actin was used as a loading control. The percentages of the immunoreactivity of the fragments relative to the total immunoreactivity per lane were calculated, and the values were expressed as arbitrary units relative to the sham group, which was assigned a value of 1. Each bar represents the mean ± SEM; *n* = 6 rats. ^#^*P* < 0.05, ^##^*P* < 0.01 vs sham. *AMC* 7-amido-4-methylcoumarin

Next, we measured calpain activity. We found that its activity immediately increased at 0.5 h and reached the highest level at 1 h (Fig. 2a). To our surprise, calpain activity remained at high levels until 24 h post-scald burn (Fig. 2a). As membrane skeleton proteins are calpain substrates, we measured the full-length and fragment levels of α-fodrin and ankyrin-B. The Western blot data showed that proteolysis levels correlated well with the tissue damage. The levels of the 145/150-kDa cleavage fragment of α-fodrin were two to three times higher at 0.5, 1, 6, and 12 h in the burn injury group than those in the sham group (Fig. 2b). The level of the 180-kDa cleavage fragment of ankyrin-B increased two to three times at the indicated time points (Fig. 2c). Finally, the proteolysis of both α-fodrin and ankyrin-B diminished at 24 h (Fig. 2b, c).

### Calpain inhibition with MDL28170 blunted scald burn-induced acute lung injury but not skin damage

**Fig. 3:**
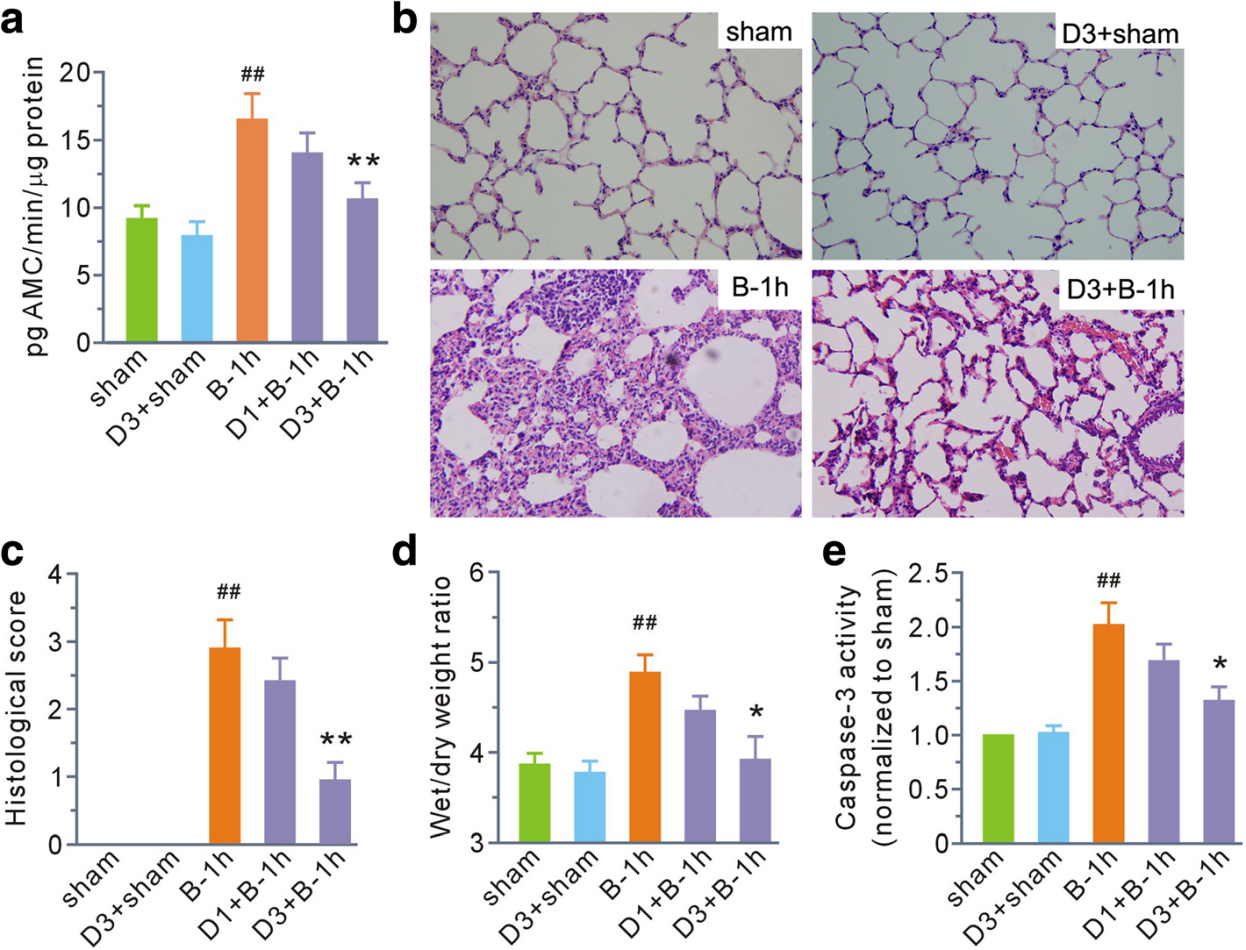
MDL28170 inhibited calpain activity in lung tissue and ameliorated lung damage in the first hour after burn injury. **a** Grouped results of calpain activity. **b** Representative hematoxylin and eosin staining of lung tissue (magnification, × 200). **c**–**e** Grouped results of histology score, wet to dry weight ratios, and caspase-3 activity, respectively. Each bar represents the mean ± SEM; *n* = 6 rats. ^##^*P* < 0.01 vs sham. *P < 0.05, **P < 0.01 vs B-1 h. B-1 h, the samples were obtained at the first hour after scald burn. D1 and D3 stand for MDL28170 at a dose of 1 and 3 mg/kg b. w., respectively. MDL28170 was given intravenously 1 h before burn injury. *AMC* 7-amido-4-methylcoumarin

We examined the effects of the calpain inhibitor MDL28170 on calpain activity and acute lung injury at 1 h post-scald burn. As shown in Fig. 3a–e, MDL28170 at a dose of 1 mg/kg b. w. had no effects on any parameter. However, when the dosage was increased to 3 mg/kg b. w., calpain activity, histological score, the wet/dry weight ratio, and the activity of caspase-3 post-scald burn were all significantly prevented. Treatment of normal rats with MDL28170 at 3 mg/kg b. w. had no effects on histological score, wet/dry weight ratio, or activity of caspase-3 compared with the sham group.

**Fig. 4:**
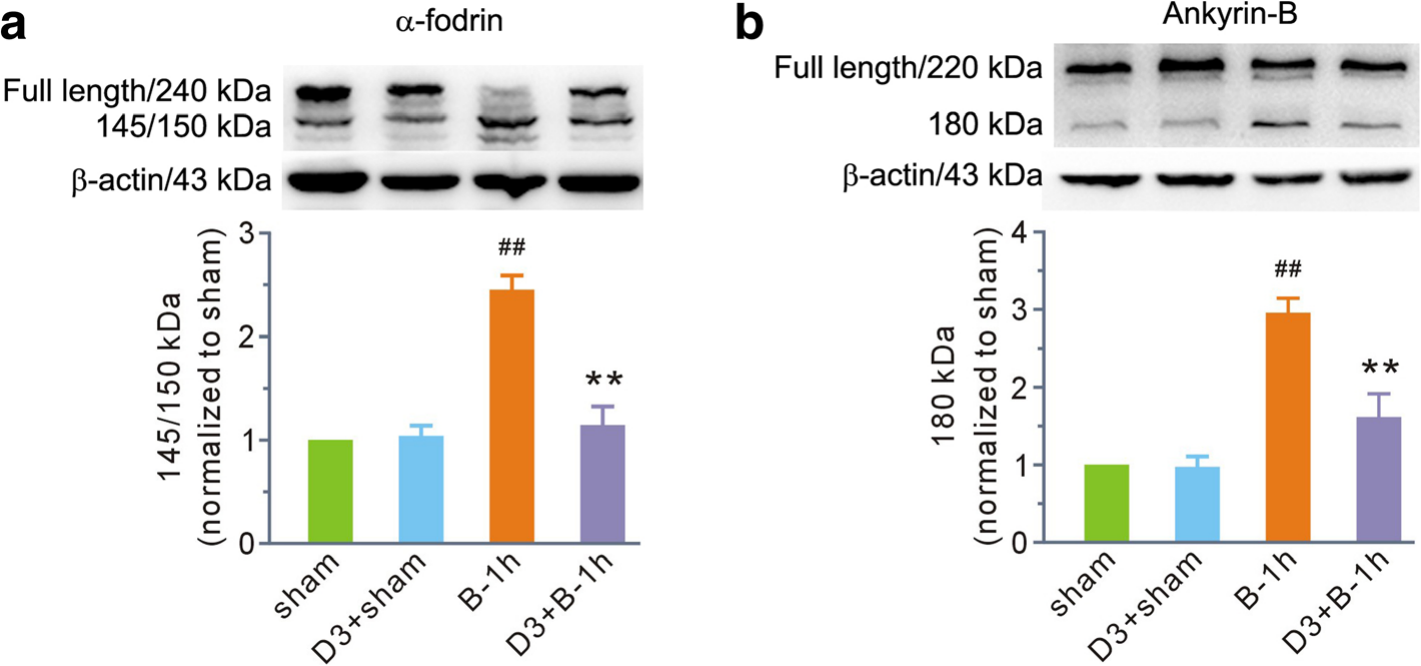
MDL28170 blunted (**a**) α-fodrin and (**b**) ankyrin-B degradation in the first hour after burn injury. The top image in each panel shows representative blots for the full-length and fragmented forms of α-fodrin and ankyrin-B. β-actin was used as a loading control. The bottom image in each panel shows the grouped results of the densitometric analysis. The values were expressed as arbitrary units relative to the sham group, which was assigned a value of 1. Each bar represents the mean ± SEM; *n* = 6 rats. ^##^*P* < 0.01 vs sham. **P < 0.01 vs B-1 h. B-1 h, the samples were obtained at the first hour after scald burn. D3 stands for MDL28170 at a dose of 3 mg/kg b. w. MDL28170 was given intravenously 1 h before burn injury

Then, we chose MDL28170 at a dose of 3 mg/kg b. w. to determine its effects on calpain-mediated membrane skeleton protein degradation at 1 h post-scald burn. MDL28170 had no effects on α-fodrin and ankyrin-B in normal rats; however, it significantly attenuated fragment levels of α-fodrin and ankyrin-B caused by scald burn (Fig. 4).

**Fig. 5:**
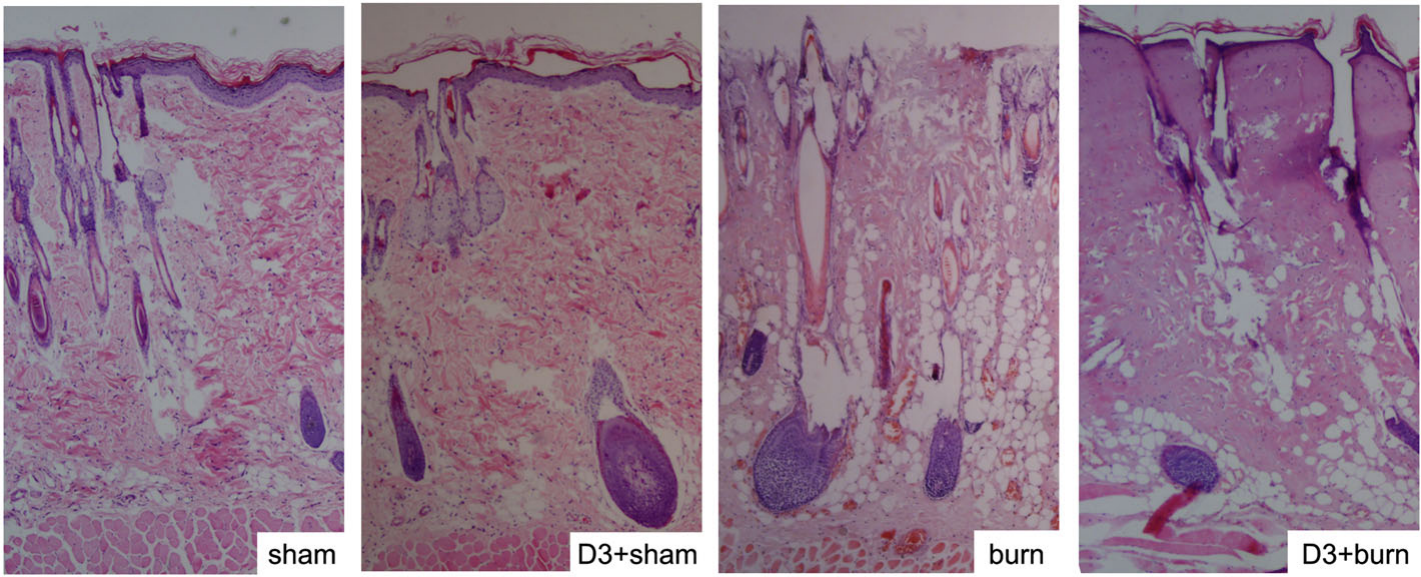
Representative microscopic skin structure images with or without MDL28170 administration (magnification, × 40). Hematoxylin and eosin staining was performed at 3 h post-scald burn. Sham, sham group; D3+sham, sham with MDL28170 group; burn, burn injury group; D3+burn, burn injury with MDL28170 group. D3 stands for MDL28170 at a dose of 3 mg/kg b. w. MDL28170 was given intravenously 1 h before burn injury

Histological examination of dorsal skin was also performed. The sham group presented a thin epidermis with corneal layer. The superficial papillary dermis showed lax connective tissue, pilous follicles, and sebaceous glands, and deep reticular dermis had collagen bundles (Fig. 5). Full-thickness skin damage in the burn group was characterized by coagulation necrosis in the epidermis, the superficial papillary dermis, and the deep reticular dermis, with homogenization of collagen fibers in deep reticular dermis (Fig. 5). MDL28170 at a dose of 3 mg/kg b. w. failed to prevent these alterations, and it had no effect in normal rats (Fig. 5).

### MDL28170 eliminated calpain 1-positive leukocytes from lung tissue after scald burn

**Fig. 6:**
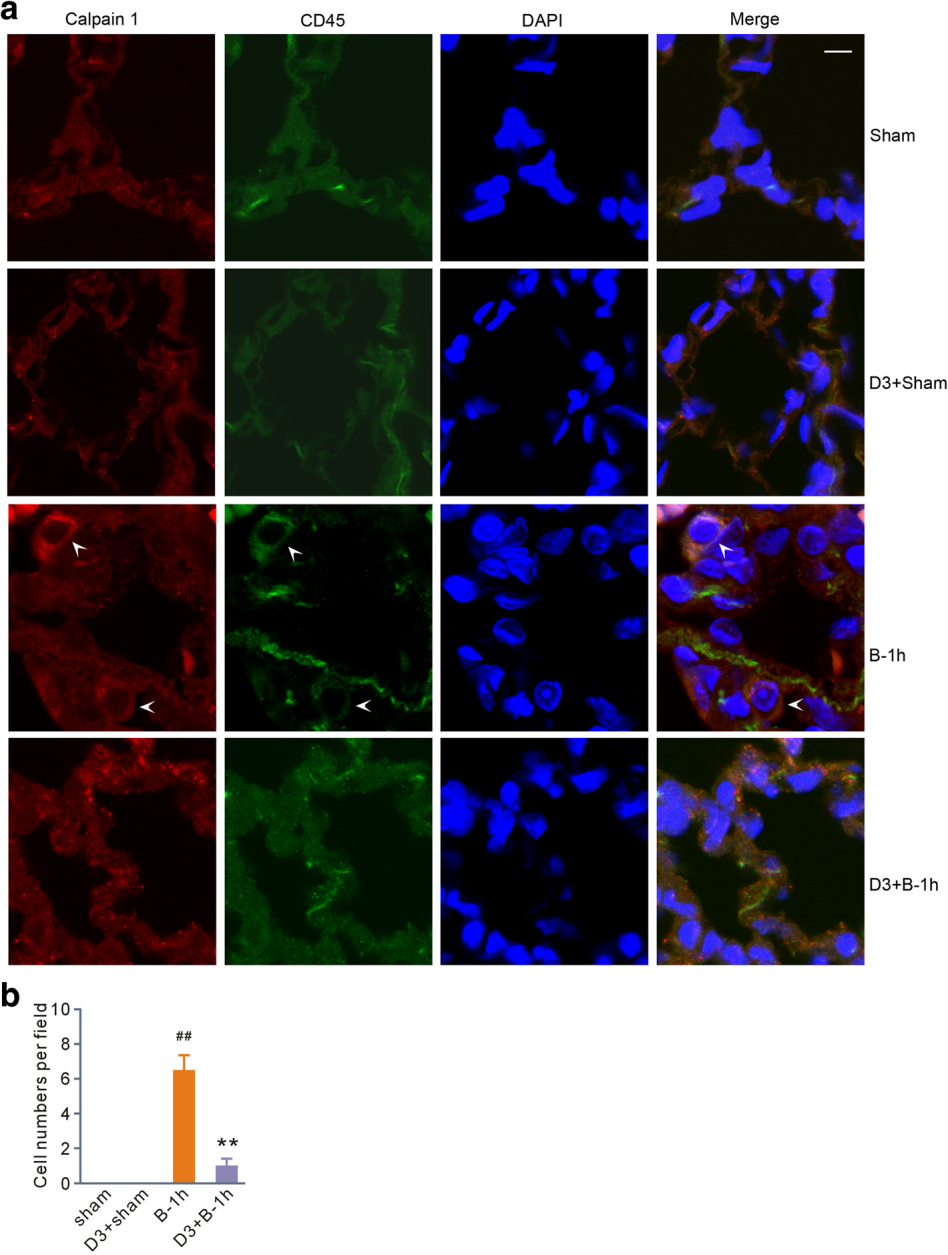
MDL28170 reduced the number of calpain 1 and CD45 double-positive cells in the lung tissue of rats exposed to scald burn injury. **a** Representative immunofluorescence images in the sham, sham with MDL28170 (D3+Sham), burn injury (B-1 h), and burn injury with MDL28170 (D3+B-1 h) groups. The nuclei were counterstained with DAPI (blue). Arrows indicate the cells positive for calpain 1 and CD45 staining. Scale bar, 5 μm. **b** Grouped results of the positive cell numbers in a field of × 600 magnification. Each bar represents the mean ± SEM; *n* = 6 rats. ^##^*P* < 0.01 vs sham. **P < 0.01 vs B-1 h. B-1 h, the samples were obtained at 1 h after scald burn injury. D3 stands for MDL28170 at a dose of 3 mg/kg b. w. MDL28170 was given intravenously at a dosage of 3 mg/kg b. w. 1 h before burn injury

Taking into consideration the finding that calpain is involved in the inflammatory process [17, 18], we performed double immunofluorescent staining for calpain 1 and the common leukocyte surface marker CD45 [21]. The confocal images at 1 h post-scald burn showed calpain 1 and CD45 double-positive cells in the lung tissue, indicating leukocytes were a dominant source of calpain (Fig. 6). More importantly, MDL28170, which had no effects in normal rats, prevented calpain 1-positive leukocytes from infiltrating the lung tissue after scald burn (Fig. 6).

### MDL28170 given at 1 h post-scald burn protected partially against scald burn-induced lung injury

**Fig. 7:**
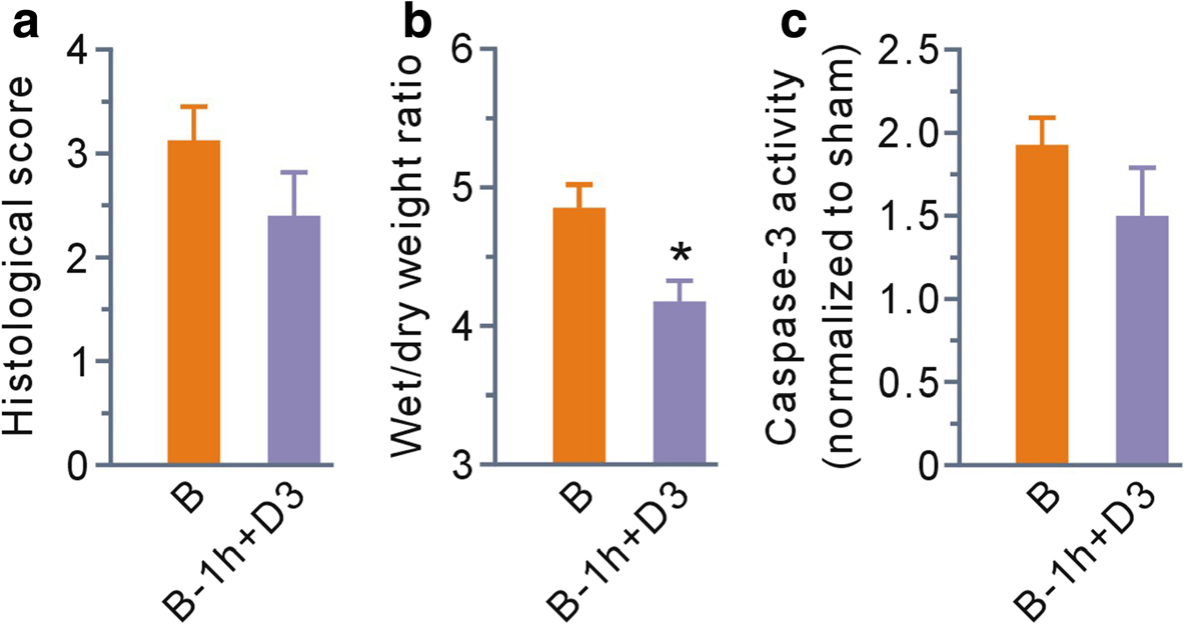
MDL28170 given at 1 h post-scald burn protected partially against scald burn-induced lung injury. **a**–**c** Grouped results of histological scores, wet to dry weight ratios, and caspase-3 activity at 6 h post-scald burn, respectively. Each bar represents the mean ± SEM; *n* = 6 rats. *P < 0.05 vs B. B, burn injury group; B-1 h+D3, the group that received MDL28170 (3 mg/kg b. w.) at 1 h post-scald burn

Finally, we examined the effects of MDL28170 given at 1 h post-scald burn on the lung injury. Treatment with MDL28170 markedly reduced wet/dry weight ratio at 6 h post-scald burn compared with the burn group (Fig. 7), although the differences of histological scores and the activity of caspase-3 between two groups had no statistical significance (Fig. 7).

## Discussion

Severe burn brings about several pathophysiological changes, including inflammatory infiltration [19, 22], oxidative stress [23, 24], and initiation of apoptosis [20], all of which contribute to acute lung injury. In this study, we demonstrated that (1) scald burn induced acute lung injury that was accompanied with an increase in calpain activity; (2) calpain inhibition with MDL28170 ameliorated the acute lung injury in intact rats, as reflected by reduced histological scores, wet/dry weight ratios, and caspase-3 activity, an indicator of cell apoptosis; and (3) calpain inhibition reduced the proteolysis of membrane skeleton proteins α-fodrin and ankyrin-B, both of which are critical for cell structure and function. These data provided the first evidence that calpain is a mediator of acute lung injury after scald burn and suggested that its destructive action involves proteolysis of membrane skeleton proteins.

Immunofluorescence images revealed calpain 1 and CD45 double-positive cells in the lungs of rats exposed to scald burn injury. These data provided evidence that leukocytes are a dominant source of calpain. As the calpain inhibitor MDL28170 gave no discernible protection against burn-induced skin damage in the epidermis and deep dermis structures, these results can be interpreted to support the view that acute lung injury is secondary to skin damage-induced inflammatory infiltration. Calpain is not only an intracellular constituent but is also released into the extracellular milieu. The latter behavior was implicated in regulation of tissue structure in acute glomerulonephritis via cleaving its substrates [25]. Taking these findings into consideration, we suggest that severe scald burn elicits leukocyte infiltration, resulting in secretion of excessive calpain, consequently destroying the lung tissue structure.

Membrane skeleton proteins take part in organizing epithelial cell shape [11], assembling epithelial junctions [12, 26], and tethering sodium channels to the apical membrane [13], all of which affect the degree of epithelial fluid transport. In this study, time-course studies revealed that the degradation of membrane skeleton proteins α-fodrin and ankyrin-B correlated well with the degree of acute lung injury. Furthermore, calpain inhibition decreased levels of membrane skeleton protein fragments and wet/dry weight ratios. We suggest that calpain destroys alveolar epithelium via degrading membrane skeleton proteins, contributing to scald burn-induced acute lung injury.

Calpain also plays a pivotal role in the inflammatory process [17, 18]. Here, the data showed that the calpain inhibitor MDL28170 decreased leukocyte infiltration, an important parameter for histological score. These results suggest that calpain enhances the inflammatory response in scald burn-induced lung injury. Currently, 15 calpain genes have been described in the human genome [6]. We only evaluated calpain 1 in this study, and the data revealed that it was involved in acute lung injury. Whether other isoforms also contribute to the injury warrants further investigation.

Instant fluid resuscitation is a critical step to reduce multiorgan failure after scald burn. It protects patients from a decrease in circulating blood volume and cardiac output [27]. Furthermore, it prevents broad-spectrum protease inhibitor leupeptin-sensitive enzyme activation caused by delayed fluid resuscitation [28]. Here, we found that the activity of calpain was upregulated in the scenario of instant fluid resuscitation and that inhibition of calpain ameliorated the lung injury. We suggest that activation of calpain is an independent factor contributing to scald burn-induced lung injury.

In this study, the calpain inhibitor MDL28170 had no discernible effects in normal rats, whereas the drug given before burn injury protected against scald burn-induced lung injury. These results suggest that calpain may be a promising candidate target in treatment of lung damage after burn injury. What is more important is that MDL28170 given at 1 h post-scald burn injury reduced lung wet/dry weight ratios compared with the burn group, although it had no obvious effects on histological score and caspase 3 activity. The data strongly support that calpain inhibition as early as possible is a potential strategy for ameliorating scald burn-induced lung injury.

Last but not least, the data showed that calpain activity remained at high levels at 24 h post-scald burn injury, a different result from the alteration of its substrates α-fodrin and ankyrin-B. Our data were consistent with the findings of Merritt [29], but not with those of Wong [30], supporting the viewpoint that continued high levels of calpain activity participate in the high energy expenditure response to burn injury in the late phase.

## Conclusions

This study is the first to provide evidence that calpain, a product of infiltrating leukocytes, is a mediator of scald burn-induced acute lung injury. Its destructive actions involve enhancement of inflammation and proteolysis of membrane skeleton proteins. Our data also support the notion that calpain has late effects in the lung after scald burn injury. Further studies on its effects and the underlying mechanisms would be helpful to assess its potential as a therapeutic target in ameliorating acute lung injury after scald burn.
